# miRNAs in mtDNA-less cell mitochondria

**DOI:** 10.1038/cddiscovery.2015.4

**Published:** 2015-07-27

**Authors:** N Dasgupta, Y Peng, Z Tan, G Ciraolo, D Wang, R Li

**Affiliations:** 1 Division of Human Genetics, Cincinnati Children’s Hospital Medical Center, 3333 Burnet Avenue, Cincinnati, OH 45229, USA; 2 Division of Pathology, Cincinnati Children’s Hospital Medical Center, 3333 Burnet Avenue, Cincinnati, OH 45229, USA; 3 Maxillofacial Pathology and Radiology Department, Ohio State University College of Dentistry, 304 W. 12th, Columbus, OH 43210, USA; 4 Department of Pediatrics, University of Cincinnati College of Medicine, Cincinnati, OH 45229 USA

## Abstract

The novel regulation mechanism in mtDNA-less cells was investigated. Very low mtDNA copy in mtDNA-less 206 *ρ*° cells was identified. But no 13 mitochondria-specific proteins were translated in 206 *ρ*° cells. Their mitochondrial respiration complexes V, III and II were 86.5, 29.4 and 49.6% of 143B cells, respectively. Complexes I and IV completely lack in 206 *ρ*° cells. Non-mitochondrial respiration to generate ATP in 206 *ρ*° cells was discovered. The expression levels of some mitochondrial RNAs including 12S rRNA, COX1, COX2, COX3, ND4 and ND5 were low. However, ND1, ND3 and Cyto b were not expressed in 206 *ρ*° cells. Unequal transcription of mitochondrial RNAs indicated the post-transcriptional cleavage and processing mechanisms in the regulation of mitochondrial gene expression in 206 *ρ*° cells. MicroRNAs (miRNAs) may modulate these mitochondrial RNA expression in these cells. RNA-induced silencing complex indeed within 206 *ρ*° cell mitochondria indicated miRNAs in 206 *ρ*° cell mitochondria. miRNA profile in mtDNA-less 206 *ρ*° cells was studied by next-generation sequencing of small RNAs. Several mitochondria-enriched miRNAs such as miR-181c-5p and miR-146a-5p were identified in 206 *ρ*° cell mitochondria. miR-181c-5p and miR-146a-5p had 23 and 19 potential targets on mitochondrial RNAs respectively, and these two miRNAs had multiple targets on mitochondria-associated messenger RNAs encoded by nuclear genes. These data provided the first direct evidence that miRNAs were imported into mitochondria and regulated mitochondrial RNA expressions.

## Introduction

MicroRNAs (miRNAs) regulate gene expression via partial base pairing within RNA-induced silencing complex (RISC) to their target messenger RNA (mRNAs) for degradation or suppression of mRNA translation. The precursors of miRNAs are small non-coding RNA molecules. miRNAs are transcribed as ~70 nucleotide precursors and subsequently processed by the RNase-III type enzyme Dicer to give a 18- to 25-nucleotide mature product. The mature miRNA is part of an active RISC containing Dicer and many associated proteins. Members of the Argonaute protein family are central to RISC function. Argonaute 2 (Ago2) is the key component of RISC.^1–3^ In general, miRNA are highly conserved in different species, and are important regulators of gene expression in human cells. An explosion of interest has led to ~2000 mammalian miRNAs being described. miRNAs have been shown to have critical roles in many cellular and disease processes.^4–7^ Several miRNAs have been shown to be imported into mitochondria and involved in the regulation of mitochondrial metabolism.^8,9^ Characterizing the expression profile of miRNAs in mitochondria is a first step toward understanding their role in mitochondrial gene regulation.

Mitochondria, supplying 90% of energy for cells, are also involved in many other essential cellular processes, such as creating reactive oxygen species, regulating apoptosis and buffering calcium.^10^ More than 1500 proteins are found in the mitochondria.^11^ The majorities of mitochondrial proteins are products of nuclear genes and are synthesized in the cytosol, then imported into the mitochondria. The mitochondrial genome in mitochondria codes for only 13 peptides/proteins, 22 tRNAs and 2 ribosomal RNAs. Thirteen peptides encoded by mitochondrial genome (mtDNA) are components of respiratory chain complexes located on the inner membrane of mitochondria.

It is believed that 143B-derived mtDNA-less cells by ethidium bromide (EtBr) treatment, 206 *ρ*° cells, have no mitochondrial genome, which could not transcript to mitochondrial RNAs and are unable to translate 13 mitochondria-specific proteins.^12^ It has not reported that the effect of miRNAs in mtDNA-less 206 *ρ*° cells. This article describes a study of miRNA profile in mtDNA-less 206 *ρ*° cell mitochondria to elucidate the regulation mechanism of miRNAs on mitochondrial RNAs in mtDNA-less cells.

## Results

It was demonstrated that miRNAs regulated gene expression by targeting mRNAs through partial base-pairing complementary sites in cytosol. It was reported that some miRNAs were present in the mitochondria of liver cells, human myoblasts and HeLa cells.^8,9,13^ But the significance and functional consequences have not been characterized. The study is to determine whether miRNAs, such as proteins, could translocate into the mitochondria and regulate mitochondrial gene expression in 206 *ρ*° cells.

### mtDNA-less, not mtDNA lack, in 206 *ρ*° cells

Usually, it is accepted that 206 *ρ*° cells is an mtDNA-lacking cell line. To detect whether 206 *ρ*° cells contain mtDNA, DNA samples from 143B and 206 *ρ*° cells were amplified in 24 overlapping fragments by use of 24 pairs of oligonucleotide primers.^14^ We found that some mitochondrial genomic regions in 206 *ρ*° including primers 2, 8, 9 11, 12, 13 and 19 (Supplementary Figure S1) had the same PCR amplicons as 143B cells. After sequencing these PCR amplicons and BLAST homology searches on National Center for Biotechnology Information (NCBI) website,^15^ we confirmed that these sequences of PCR products from 206 *ρ*° were same as 143B mtDNA genome including mitochondrial ribosome (12S rRNA and 16S rRNA) genes, and genes of ND2, COX1, COX2, A6 and ND5.

To determine the relative DNA copy numbers of 206 *ρ*° cells to 143B cells, qPCR was carried out to determine the ratio of mtDNA HV1 region to nucleus-encoded *H3* gene. The results demonstrated that mtDNA copy numbers in 206 *ρ*° was 1/164 of mtDNA in 143B cells.

### Partial expression of mitochondrial RNAs

**Figure 1:**
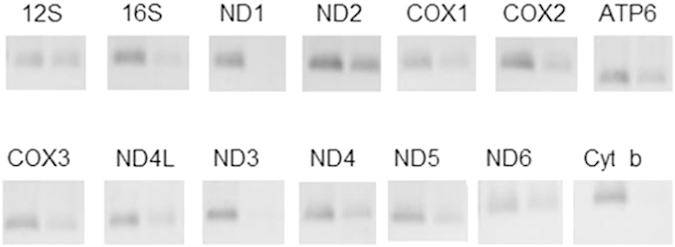
The assay of mitochondrial RNA expression efficiency by RT-PCR. Left bands are from 143B mt-R RNA cDNA and right bands are from 143B-206 *ρ*° mt-R cDNA.

To examine whether 206 *ρ*° mtDNA transcript mitochondrial mRNA, RT-PCR was performed using cDNA from 206 *ρ*° mt-R RNA sample. The results demonstrated that some mitochondrial RNAs were expressed in 206 *ρ*° cells (Supplementary Figure S1) including 12S and 16S rRNA, ND2, COX1, COX2, A6, ND4, ND4L, ND5 and ND6 RNAs. But the expression levels of these mitochondrial RNAs were lower than in 143B cells. And mitochondrial mRNAs of ND1, ND3 and Cytb were not expressed in 206 *ρ*° cells (Figure 1). RT-qPCR results confirmed that the expression levels of ND1, ND3 and Cyto b mRNAs were extremely low.

### Lack of mitochondria-specific protein synthesis

The mitochondrial genome encodes for 37 genes: 2 rRNAs (12S and 16S rRNA) and 22 tRNAs required for mitochondrial protein synthesis and 13 mitochondria-synthesized peptides essential to the respiratory chain complexes. The results of [^35^S]-pulse-labeling mitochondrial proteins *in vitro* demonstrated that 206 mtDNA-less *ρ*° cells did not translate 13 mitochondria-specific peptides/proteins (Supplementary Figure S2), even though some mitochondrial genomic regions were detected and some mitochondrial mRNAs were partially expressed in 206 *ρ*° cells.

### Decreased expression of mitochondrial complexes

Oxidative phosphorylation is an important cellular process that uses oxygen and simple sugars to produce ATP. Five protein complexes (complexes I–V), made up of several proteins encoded by nuclear gene or/and mitochondrial genomic genes, are involved in this process. The protein ratios of mitochondrial genomic gene encoded to unclear gene encoded in complexes I–V are 7/37, 0/4, 4/25, 3/16 and 2/17, respectively. Our western blot results showed the significantly decreased amounts of complexes II (29.4%) and III (49.6%) compared with 143B cells, and complexes I and IV lacked completely in 206 *ρ*° cells (Supplementary Figure S3). The antibody to probe complex IV is anti-COX1. Complex IV completely lost in western blotting confirmed no mitochondrial COX1 were synthesized in 206 *ρ*° cells by ^35^S pulse labeling of mitochondrial protein synthesis even though partially expressed COX1 mRNA (Figure 1). But complex V kept high expression in 206 *ρ*° cells. The expression of complex V in 206 *ρ*° cells was 86.5% when compared with that of 143B cells.

### Marked decreased mitochondrial respiration

**Figure 2:**
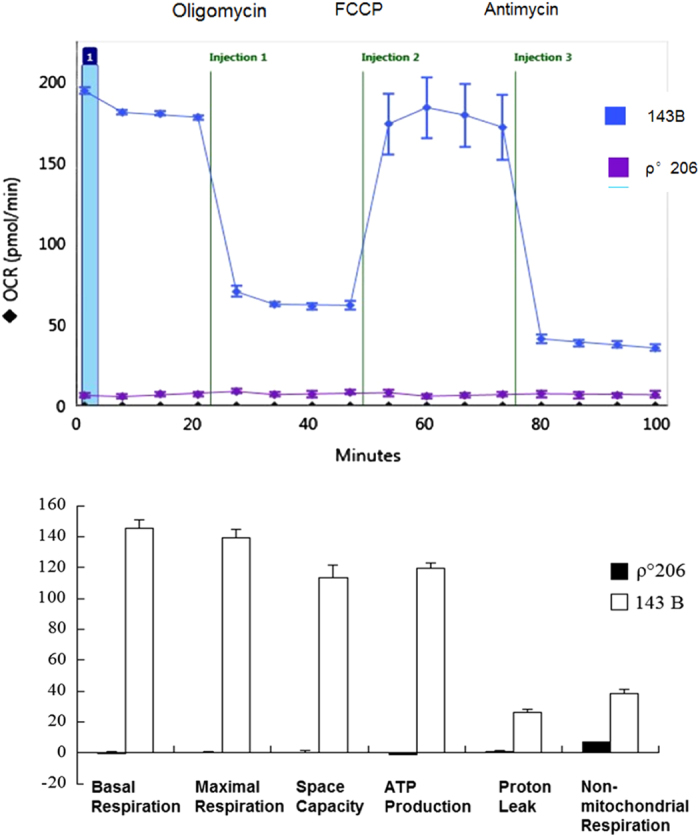
Mitochondrial function assay of 143B and 143B-206 *ρ*° cells by Seahorse XF 24 analyzer. The bioenergetic profiles of 143B and 143B-206 *ρ*° cells were generated by injecting electron transport chain inhibitors: oligomycin, FCCP and antimycin A to assess mitochondrial respiration. The results indicated mitochondrial dysfunction in 143B-206 *ρ*° cells. Non-mitochondrial respiration was kept in 143B-206 *ρ*° cells.

No mitochondria-specific 13 peptides/proteins were synthesized, and the expression levels of mitochondrial respiration complexes were decreased in 206 *ρ*° cells. We supposed that mitochondrial respiration dysfunction would be exhibited in 206 *ρ*° cells. The Seahorse assay^16^ results demonstrated completely reduced respiratory chain function and increased non-mitochondrial respiration in 206 *ρ*° cells (Figure 2). This result indicated that 206 *ρ*° cells produced ATP that primarily relied on non-mitochondrial respiration after mitochondrial dysfunction.

### Mitochondrial morphological alterations

Mitochondrial biochemical abnormalities in 206 *ρ*° cells were confirmed. We reasoned whether the presence morphological alterations in 206 *ρ*° cells. Transmission electron microscopy (TEM) images showed that the numbers of mitochondria were increased in 206 *ρ*° cells, the size of mitochondria was smaller and their mitochondria exhibited that the cristae was broken, internal membranes were disorganized, outer membrane was clumped and detached, and mitochondrial matrix was reduced with large empty vacuoles in 206 *ρ*° cells (Supplementary Figure S4).

### The presence of Ago2 within the mitochondria

**Figure 3:**
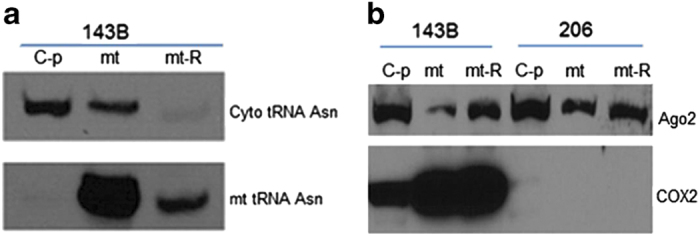
Pure mitochondria, Ago2 within mitochondria. (**a**) Northern blotting was performed by using mt tRNA Asn probe and cytosolic tRNA Asn probe to check C-p, mt and mt-R, which indicated that mitochondria were pure, and cyto tRNA was not present in mt-R. (**b**) Western blot results confirmed the presence of Ago2 within 143B and 206 *ρ*° mitochondria (mt and mt-R samples).

It was reported that various miRNAs were detected in isolated mitochondria from rat liver, rat ventricular myocytes and several common human cell lines,^9^ we anticipated that the increased miRNAs, targeting to the mitochondrial RNAs, might account for decreased translation in 206 *ρ*° cell mitochondria. To begin exploring these possibilities, we first used highly purified mitochondria (Figure 3a) to demonstrate whether a fraction of Ago2 was present within the mitochondria. The results demonstrated that Ago2 was indeed within mt and mt-R from 143B and 206 *ρ*° cells (Figure 3b). Ago2 was detected to be highly expressed in 206 *ρ*° mt and mt-R samples (Figure 3b).

### miRNA profile of mtDNA-less cells

The key component of RISC, Ago2, was within cell mitochondria of 143B and 206 *ρ*° cells. RISC should be within their mitochondria and regulate mitochondrial RNAs. Several miRNAs were detected in mitochondria.^8,9,13^ However, the functional significance of miRNAs in the mitochondria has remained largely unknown, especially in mtDNA-less cells.

#### Mitochondria-enriched miRNAs

To determine whether miRNAs were in mitochondria of 143B and 206 *ρ*° cells, we performed next-generation sequencing of small RNA (sRNA) using their mt-R RNA samples, their C-p RNA and mt RNA samples were as control. sRNA patterns of C-p RNA and mt-R RNA from 143B and 206 *ρ*° cells were shown in Supplementary Figure S5. By comparing mt-R miRNA patterns (Li-30=143B mt-R; 206 *ρ*° mt-R) with C-p miRNA patterns (Li-29=143B C-p; Li-27=206 *ρ*° C-p), we found that miRNAs in mt-R RNA samples were much less than in C-p RNA samples. The results indicated that miRNAs on outer membrane of mitochondria were digested by RNase A, and the miRNAs from mt-R should be in mitochondria. The miRNA percentages of C-p RNA samples from 143B and 143B-206 *ρ*° cells were higher than the respective mt-R RNA samples (Supplementary Figure S6). Owing to the extremely low amount of miRNAs in mt-R RNA from 206 *ρ*° cells, we performed sRNA sequencing using 206 *ρ*° mt RNA sample in order to identify mitochondria-enriched miRNAs.

To identify mitochondria-enriched miRNAs, the miRNA ratios of mt-R RNA/C-p RNA from 143B and the miRNA ratios of mt-R RNA/ mt RNA from 206 *ρ*° cells were calculated. The results demonstrated that some mature miRNAs were enriched in 143B mitochondrial RNA and 206 *ρ*° mitochondrial RNA. Thirty-nine miRNAs in 206 *ρ*° mt-R RNA were enriched compared with 206 *ρ*° mt RNAs including miR-133a, 499a-5p, 372, 1, 127-3p, 126-3p, 1305, 501-3p, 143-3p, 887, 122-5p, 491-5p, 146a-5p, 181c-5p and 145-5p and so on (Supplementary Table S7). Twenty-eight miRNA in 143B mt-R RNA were higher than C-p RNA including miR-4787-3p, 4510, 141-3p, 203, 200c-3p, 660-5p, 181d, 146a-5p, 181c-5p, 99a-5p, 365a-3p, 365b-3p, 138-5p, 501-3p, 342-3p, 193b-3p, 99b-3p, let-7c, 181a-2-3p, 103a-3p, 19b-3p, 10b-5p, 125a-5p, 484, 148b-3p and 181a-5p (Supplementary Table S7).

**Figure 4:**
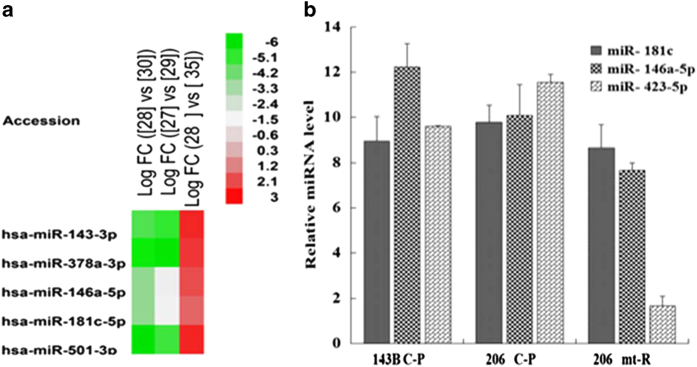
(**a**) Hotmap of mitochondria-enriched miRNAs. 30=143B mt-R RNA, 28=206 *ρ*° mt-R RNA, 29=143B C-p RNA, 27=206 *ρ*° C-p RNA and 35=206 *ρ*° mt RNA. (**b**) RT-qPCR results confirmed miR-181c-5p (miR-181c), miR-146a-5p were highly enriched in 206 *ρ*° mitochondria (mt-R), the control miR-423-5p was not enriched in 206 *ρ*° mitochondria. 5S was as an internal control for quantification.

From the miRNA ratios of 206 *ρ*° mt-R/143B mt-R, 206 *ρ*° C-p/143B C-p and 206 *ρ*° mt-R/206 *ρ*° mt, we found that miR-143-3p, 378a-3p, 146a-5p,181c-5p and 501-3p were presented in RNA samples of 206 *ρ*° C-p, 206 *ρ*° mt, 206 *ρ*° mt-R, 143B C-p and 143B mt-R, respectively (Figure 4a and Supplementary Table S7). These five miRNAs were more enriched in 206 *ρ*° mt-R RNA than in 206 *ρ*° mt RNA (Figure 4a and Supplementary Table S7). High-expression levels of miR-181c-5p and 146a-5p in 206 mt-R RNA indicated that miR-181c-5p and 146a-5p might modulate mitochondrial RNAs in these cells.

#### miRNAs target on mitochondria-specific RNAs

Computational analyses of miRNA targets showed that miR-181c-5p had 23 and miR-146a-5p had 19 potential targets on mitochondrial mRNAs, rRNAs, tRNAs and non-coding regions (Table 1). The results indicated that miR-181c-5p and miR-146a-5p may inhibit mitochondrial RNAs to modulate mitochondrial gene expression.^8,9,17^

To determine whether miR-181c-5p and miR-146a-5p were enriched in 206 *ρ*° cell mitochondria, we also performed RT-qPCR using miRNA cDNAs from 143B C-p RNA, 206 ρ° C-p RNA and 206 *ρ*° mt-R RNA to validate our sRNA-sequencing data. The results demonstrated that miR-181c-5p and miR-146a-5p were highly enriched in 206 *ρ*° mitochondrial fraction compared with control miR-423-5p (Figure 4b).

#### miRNAs target on mitochondria-associated mRNAs encoded by nucleus

More than 1500 genes are associated with mitochondrial structure and/or function.^11^ The majorities of these genes are encoded by nucleus. miRNAs may target on mitochondria-associated RNAs encoded by nuclear genes to regulate mitochondrial metabolism and mitochondrial function. There are 892 predicted targets for hsa-miR-181c-5p and 224 predicted targets for hsa-miR-146a-5p in miRNA database. Some targets are mitochondria-associated mRNAs, such as miR-181c-5p targets on SLC25A37, SLC7A11, SLC4A8, SLC4A10, MICU3, GPD1L, KCNQ5 and CAMK2D. miR-146a-5p targets on SLC10A3, SLC38A1, SCN3B, KCNJ16 (potassium inwardly rectifying channel, subfamily J, member 16), KCNA6 and CASK and so on.

miR-181c-5p and miR-146a-5p were mitochondria-enriched miRNAs in 206 *ρ*° mitochondria. However, the expression levels of some miRNAs were low in 206 *ρ*° mitochondria. From miRNA ratios of 206 *ρ*° mt-R/206 ρ° mt RNA, we found that low-expressed miRNAs in 206 *ρ*° cell mitochondria included miR-155-5p, 196-5p, 128, 148b-3p, let-7b-5p, let-7a-5p, 197-3p,18a-5p, 15b-5p and so on (Supplementary Table S8). These miRNAs had targets on complex V component mRNAs such as miR-155-5p targeted on ATP2C1, miR-128 on ATP8A1, miR-148b-3p on ATP11A and ATP8A1, let-7b-5p and let-7a-5p on ATP2B2, ATP2B4 and ATP2C2, miR-197-3p on ATP6V1A, miR-18a-5p on ATP2C2 and ATP8A1, and miR-15b-5p on ATP13A3 and ATP1B4 mRNA. In the meantime, several miRNAs targeted on mRNAs related to mitochondrial fission, fusion or RISC proteins, including miR-196a-5p targeted on OPA1 and OPA3, miR-18a-5p on HSF2 and miR-15b-5p on HSPA4L mRNA. These low-expressed miRNAs in 206 *ρ*° mitochondria would ameliorate the inhibition of miRNAs on mitochondria-associated mRNAs, and benefit for ATP production and mitochondrial dynamic change.

## Discussion

### Mitochondrial dysfunction and compensation

EtBr has been shown to have effects on mammalian mtDNA replication. Relatively low concentrations of EtBr (0.1–2 *μ*g/ml) result in either partial or complete inhibition of mtDNA replication, but have no effect on the replication of nuclear DNA.^12^ The mtDNA-less 206 *ρ*° cells were derived from 143B cell line using low concentration of EtBr.^12^ We found low mtDNA copy number and partial expression of mitochondrial RNAs in mtDNA-less 206 *ρ*° cells. But no 13 mitochondria-specific peptides were synthesized in 206 *ρ*° cells. Compared with 143B, mitochondrial complexes V, III and II were 86.5, 29.4 and 49.4% respectively, and complexes I and IV were completely lost in 206 *ρ*° cells. No mitochondrial ND1 and ND3 mRNAs expressed in 206 *ρ*° cells may account for their loss of complex I. No mitochondrial cyto b mRNA may cause their complex III to decrease. Low expression of COX1, COX2 and COX3 may result in complex IV loss.

It is possible to maintain human cells that lack mtDNA, and thus lack mitochondrial respiration. Because of the absence of respiratory chain, 206 *ρ*° cells develop growth requirements for pyrimidines and pyruvate. Uridine is a pyrimidine nucleotide that is required for synthesis of RNA and DNA.^18^ If there is no uridine in the culture medium, mtDNA-less 206 *ρ*° cells will die in 4 days. Our results from Seahorse indicate that the respiration of mtDNA-less 206 *ρ*° cells is non-mitochondrial respiration. These cells produce ATP from pyruvate, from glycolysis of glucose and from medium supplement to generate acetyl-CoA into Krebs cycle in the matrix of mitochondria. Therefore, uridine and pyruvate in culture medium are necessary supplements for mtDNA-less cell growth.

Another reason to maintain the growth of mtDNA-less 206 *ρ*° cells is compensation mechanisms. The first is the increased mitochondria numbers in 206 ρ° cells (Supplementary Figure S4) to increase respiration volume. Also, high-activity complex V (86.5% of 143B cell) benefits to generate ATP efficiently.

### miRNAs target on mitochondrial mRNAs

Mitochondrial RNAs of ND1, ND3 and cyto b did not express in 206 *ρ*° cells, whereas the expression of other RNAs including 12S, 16S, ND2. COX1, COX2, ATP6, COX3, ND4L, ND4, ND5 and ND6 was low in 206 *ρ*° cells compared with 143B cells. The decreased mitochondrial RNA expression is the result from low copy of mtDNA in 206 *ρ*° cells (mtDNA is 1/164 of 143B cells). But unequal transcription of mitochondrial RNAs (Figure 1) may indicate the post-transcriptional cleavage and processing mechanisms in the regulation of mitochondrial gene expression.^19^ miRNAs, as important post-transcriptional regulators of gene expression in human cells, may modulate these mitochondrial RNAs expression.^8,9^ It is confirmed that several miRNAs are translocated into mitochondria and modulate specific mitochondrial genome-encoded transcripts.^8,9^ The mature miRNA is incorporated into RISC, which recognizes target mRNAs through imperfect base pairing with the miRNA, and most commonly results in translational inhibition or destabilization of the target mRNA. Ago2 is highly expressed in 206 *ρ*° cell mitochondria, which indicate that RISC is within 206 *ρ*° cell mitochondria, and miRNAs regulate mitochondrial RNAs.

sRNA sequencing and qPCR analysis data confirmed that miR-181c-5p and miR-146a-5p were enriched in 206 *ρ*° cell mitochondria. These two miRNAs have multiple targets on mitochondrial RNAs (Table 1). miR-181c-5p has 23 potential targets on mitochondrial RNAs including 12S rRNA, 16 S rRNA, ND1, ND4, ND5, ND6, COX1-3, ATP6 and Ctyb mRNA. miR-181c has been confirmed to be imported into rat mitochondria and repressed rat mitochondrial COX1 expression. miR-181c has multiple potential targets (Table 1), except COX1 mRNA, and may target other mitochondrial mRNAs, rRNAs and tRNAs.

miR-146a-5p has 19 potential targets on mitochondrial RNAs including 16S rRNA, ND1, ND2, ND4, ND5, ND6 and Cytb mRNAs (Table 1). RT-PCR showed that several mitochondrial RNAs were partially expressed in 206 *ρ*° cells, but 13 of mitochondria-specific protein in 206 ρ° cells are lack. Lack of 13 of mitochondrial mRNA translation may result from mitochondrial ribosomal RNA (16S rRNA and/or 12S rRNA) dysfunction or due to miRNAs (for example miR-181c-5p and miR-146a-5p) cut mitochondrial mRNAs or suppress mitochondrial mRNA translation.

miRNAs may target on mitochondria-associated nuclear mRNAs. Both miR-181c-5p and miR-146a-5p target on SLC mRNAs. SLC25A has important role in mitochondrial fission. These two miRNAs target on ion channel and calcium transfer mRNAs encoded by nucleus, such as KCNQ5, KCNJ16, KCNA6, SCN3B, CAMK2D and CASK. The increase of miR-181c-5p and miR-146a-5p may downregulate these mitochondria-associated nuclear mRNAs to maintain the stabilization of mitochondrial membrane potential and calcium balance.

However, the expression of several miRNAs including miR-155-5p, 196-5p, 128, 148b-3p, let-7b-5p, let-7a-5p, 197-3p,18a-5p and 15b-5p was low in 206 *ρ*° mitochondria. These miRNAs were inhibitors of ATPase and mitochondrial fission and fusion mRNAs. Low expression of these miRNAs would improve the efficiency of ATP generation and mitochondria dynamic change. Non-mitochondrial respiration and the increase in the numbers of mitochondria in 206 *ρ*° may be the result of these low-expressed miRNAs.

This is the first study to investigate the molecular, metabolic and morphological abnormalities, as well as miRNA profile in mtDNA-less 206 *ρ*° cells. Several mitochondria-enriched miRNAs (including miR-181c-5p and miR-146a-5p) were identified in 206 *ρ*° cell mitochondria. These two miRNAs targeted on multiple mitochondria-specific RNAs including mitochondrial mRNA, rRNA and tRNAs, as well as on nucleus-encoded mitochondria-associated mRNAs. This study provided direct evidence that miRNAs were imported into mitochondria and regulated mitochondrial RNAs in mtDNA-less cells.

## Materials and Methods

### Cell lines

143B cells were grown in DMEM (Invitrogen) containing glucose (4.5 g/l), completed with 10% fetal bovine serum, 0.1 mg/ml sodium pyruvate and 100 U/ml penicillin–streptomycin. mtDNA-less 206 *ρ*° cells were cultured with same medium supplement with 50 *μ*g/ ml urine. Cell pellets (C-p) were collected from one 10-cm dish of 143B and 206 *ρ*° cells, then washed two times by PBS.

### Analysis of mitochondrial genome

Genomic DNA was isolated from 143B C-p and 206 *ρ*° C-p samples. The entire mitochondrial genome of 143B and 206 *ρ*° cells was PCR-amplified in 24 overlapping fragments by using sets of the light- and the heavy-strand oligonucleotide primers, as described previously.^14,20^ These PCR amplicons were purified and subsequently analyzed by direct sequencing. The BLAST homology searches were performed using the programs available on the NCBI website.^15^ The relative DNA copy numbers of 206 *ρ*° cells to 143B cells was performed by qPCR. The ratio of mtDNA HV1 region to nucleus-encoded *H3* gene was determined as the relative DNA copy numbers of 206 *ρ*° cells. PCR primers for amplification of *H3* and *HV1* genes were listed in Supplementary Table S9.^9^

### Examination of mitochondria purity and Ago2

143B and 206 *ρ*° cellular mitochondria were isolated with the Mitochondrial Isolation kit (Miltenyi Biotec, MACS, Auburn, CA, USA) and monoclonal anti-TOM22-conjugated microbeads, as described, with several modifications.^21^ Theses mitochondrial pellets (called mt) were treated with RNase A treated (mt-R) for mitochondrial RNA isolation. After determination of mitochondrial purity, we used these samples to examine whether Ago2 was present within mitochondria from 143B and 206 *ρ*° cells by western blotting using monoclonal Anti-Ago2 antibody (SAB4200085, Sigma-Aldrich, St. Louis, MO, USA).

### Analysis of mitochondrial RNA expression

RNA preparations from 143B and 206 *ρ*° C-p, mt and mt-R were obtained by using the Totally RNA kit (Ambion, Austin, TX, USA).^22,23^ RNAs (mRNA and miRNA) were extracted using the Ambion mirVana miRNA Isolation Kit (Ambion, AM1560) following manufacturer’s instruction. First-strand cDNA was synthesized from 1 *μ*g of total RNA using miScript II RT kit (Qiagen, Valencia, CA, USA; 218161) for miRNA cDNA and mRNA cDNA, according to manufacturer’s instructions. RT-qPCR (QuantiFast SYBER Green PCR kit, Qiagen, 204054) was carried out in Applied BioSystems 7500 Real Time PCR machine Applied Biosystems (Foster City, CA, USA) for quantification of mRNAs and miRNAs using these RNA samples from 143B and 206 *ρ*° cell lines. 143B mitochondrial RNA expression was used as control. The mitochondria-specific primers for RT-PCR and RT-qPCR were listed in Supplementary Table S10.

### Assay of mitochondrial proteins

The reliable method to determine whether 206 *ρ*° cells translate mitochondrial peptides is to examine mitochondrial specific 13 peptides using [^35^S]-methionine–[^35^S]cysteine pulse labeling of mitochondrial protein synthesis *in vitro* after blocking cytoplasmic protein synthesis by emetine as we described.^22,23^ Cellular protein samples from mtDNA-less 206 *ρ*° cell line and controls of 143B cells and lymphoblastoid cell line were used.

To assay the mitochondrial complexes I–V in 206 *ρ*° cells, SDS-PAGE and western blotting of 143B and mtDNA-less 206 *ρ*° cell homogenates (20 *μ*g of protein), samples were probed for mitochondrial respiratory chain complexes via total human OXPHOS WB antibody cocktail (Abcam, Cambridge, MA, USA; ab11041).

### Examination of mitochondrial morphology

C-p samples from 143B and 206 *ρ*° cells were fixed for 24 h using fixative (2% glutaraldehyde (pH 7.2) and 0.1 M cacodylate buffer (pH 7.4)), then the samples were sent to Pathology Department in CCHMC to treat with 1–2% osmium tetraoxide (OsO4), dehydrated, embedded in resin, sliced into ultra-thin sections, double-stained with uranyl acetate and lead citrate, and finally performed TEM examination.

### Measurements of mitochondrial respiration

The cultured 143B and 206 *ρ*° cells were transferred to XF96 assay plate at 10,000 cells per well and were allowed grow overnight. ATP production and oxygen consumption were determined by XF96 Extracellular Flux Analyzers (Seahorse Biosciences, North Billerica, MA, USA). The parameters included basal respiration, ATP production, maximal respiratory capacity and proton leak. The respiration parameters were normalized by cell numbers.^16^

### sRNA sequencing

RNA samples from 143B and 206 *ρ*° C-p, mt and mt-R were obtained by using the Totally RNA kit (Ambion).^22^ RNA purity was detected by our designed probes using C-p RNA, mt RNA and mt-R RNA samples. After the determination of purity of mt-R RNA from mitochondria, these RNA samples were studied by next-generation sequencing of sRNA to examine mitochondria-enriched miRNAs in 143B and 206 *ρ*° mitochondria. Next-generation sequencing of sRNA (15–49 bp), including miRNAs (18–23 bp), was carried out in The Genetic Variation and Gene Discovery Core in CCHMC (Cincinnati, OH, USA) using 500 ng C-p RNA of 143B and 206, as well as their mt and mt-R RNA samples.

### Computational analyses of miRNA targets

To scan miRNA’s potential target sites on the mitochondrial RNAs, we performed a BLAST search^15^ of mitochondrial genome sequence and mature sequences of miR-181c-5p or miR-146a-5p. In addition, we used the target Scan algorithm (http://www.targetscan.org) to search for the presence of conserved target sites that match the seed region of miR-181c-5p and miR146a-5p, and assessed the structural accessibility of the predicted target site.^24^ The targets of miR-181c-5p, 146a-5p on nucleus-encoded mitochondrial genes were obtained by searching miRNA database (http://www.mirbase.org).

### Verification of mitochondria-enriched miRNAs

To determine whether miR-181c-5p and miR-146a-5p enriched in 206 *ρ*° cell mitochondria, the miRNA cDNA synthesized from 143B C-p RNA, 206 *ρ*° C-p RNA and 206 *ρ*° mt-R RNA samples were used to do RT-qPCR to quantify miR-181c-5p and miR-146a-5p in C-p and in mitochondria. miR-423-5p was used as control.

## Figures and Tables

**Table 1:** miRNAs’ potential targets on mitochondrial rRNAs, mRNAs, tRNAs and non-coding regions

**mt Gene**	**miR-181c-5p**	**miR-146a-5p**
12S RNA	978–984	
	1229–1235	
16S RNA	2320–2326	2245–2251
		2361–2367
ND1	3593–3599	3309–3316
	3599–3605	4120–4126
	4191–4197	
ND2		4594–4600
		5044–5050
		5198–5205
		5274–5280
ND4	11 083–11 090	11 889–11 895
	11 282–11 288	
	12 013–12 019	
ND5	12 840–12 846	12 528–12 534
	13 443–13 450	12 681–12 688
	13 495–13 501	
	13 635–13 641	
ND6	14 199–14 205	14 517–14 523
	14 401–14 407	14 538–14 544
COX1	6273–6282	
COX2	7915–7921	
COX3	9425–9431	
ATP6	8976–8984	
ATP8		8516–8522
Cytb	14 910–14 916	
tRNA Ala		5615–5621
tRNA Glu		4347–4353
tRNA Gly	10 041–10 049	
tRNA Ser(UCN)		7492–7498
tRNA Ser(AGY)		12 220–12 226
D-loop	245–252	16 364–16 371
	16 066–16 072	

miR-181c-5p had 23 and miR-146a-5p had 19 potential targets.
