# Supplementary material for: miRNAs in mtDNA-less cell mitochondria
Source: Cell Death Discov. 2015 Jul 27;1:15004–. doi: 10.1038/cddiscovery.2015.4 (PMC4979498; doi:10.1038/cddiscovery.2015.4)

**S1**. 24 PCR amplicons from 143B cell DNA and some PCR amplicons from 143B-206 ρ^o^ cell DNA.


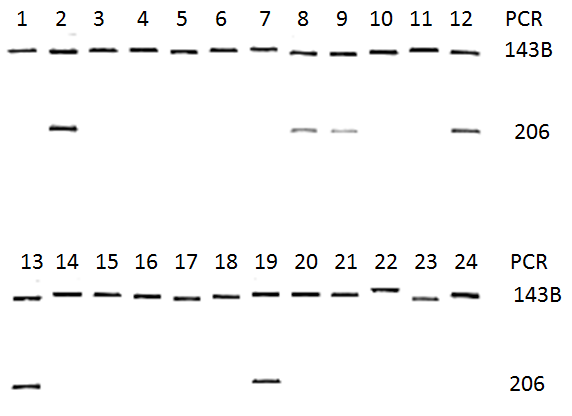


**S2**. Mitochondrial protein synthesis in a lymphoblastoid cell line (Left), 143B (Middle), 143B-206 ρ^o^ (Right) cells. There were 13 mitochondrial peptides/proteins in lymphoblastoid cell line and 143B cells. No 13 mitochondria-specific peptides/proteins were translated in 143B-206 ρ^o^ cells.


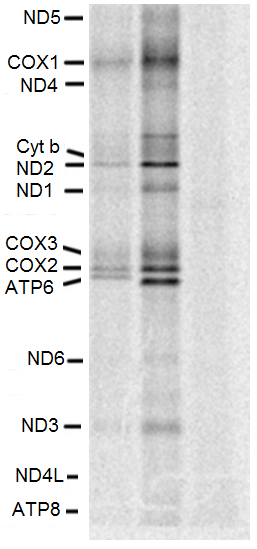


**S3.** The results of mitochondrial respiration complex I –V by Western blotting. GAPDH was as control.


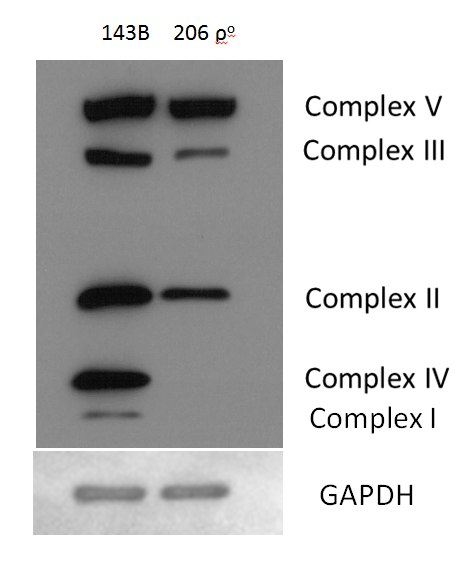

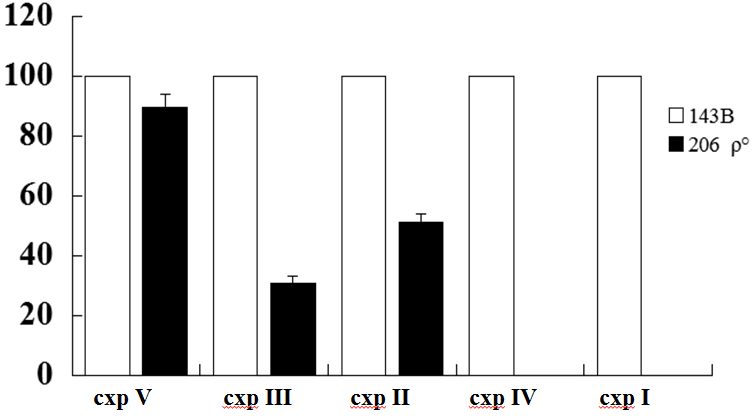


**S4.** Examination of mitochondrial morphology in 143B-206 ρ^o^ cells by TEM. Left is 143B subcellular structure. Right is 143B-206 ρ^o^ subcellular structure. Mitochondria were smaller in 143B-206 ρ^o^ cells than in143B cells. But mitochondria numbers in 143B-206 ρ^o^ were more. These mitochondria exhibited the cristae broken, internal membranes disorganized, clumped and detached with outer membrane. Mitochondrial matrix was reduced with large empty vacuoles.


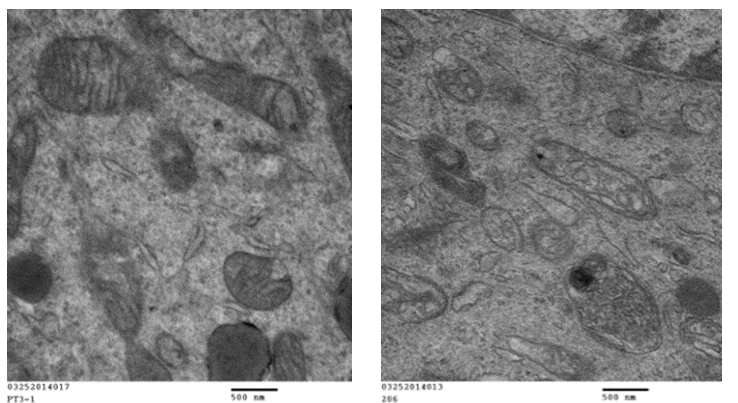


**S5.** sRNA patterns of 143B cells and 143B 206 ρ^o^ cells. Li-30=143B mt-R RNA; Li-28=206 ρ^o^ mt-R RNA; Li-29=143B C-p RNA; Li-27=206 ρ^o^ C-p RNA.


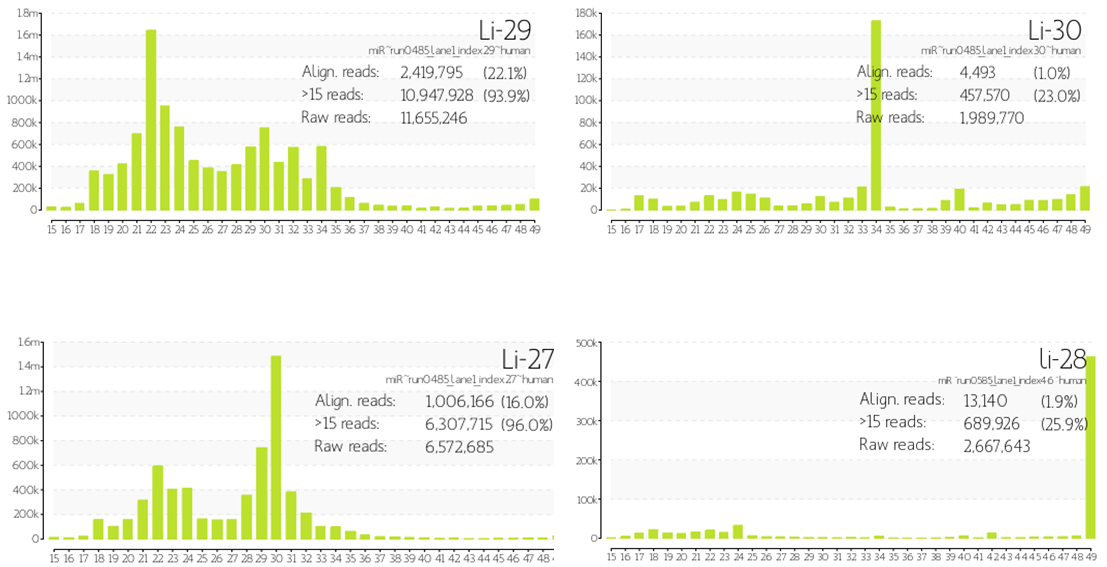


**S6.** miRNA percentages in sRNAs of 143B C-p RNA and 143B mt-R RNA, 206 ρ^o^ C-p RNA and 206 ρ^o^ mt-R RNA samples. Gray color presented miRNAs. The results indicated that C-p RNAs contained more miRNAs than in mt-R RNAs in 143B cells and 206 ρ^o^ cells. Li-30=143B mt-R RNA; Li-28=206 ρ^o^ mt-R RNA; Li-29=143B C-p RNA; Li-27=206 ρ^o^ C-p RNA.


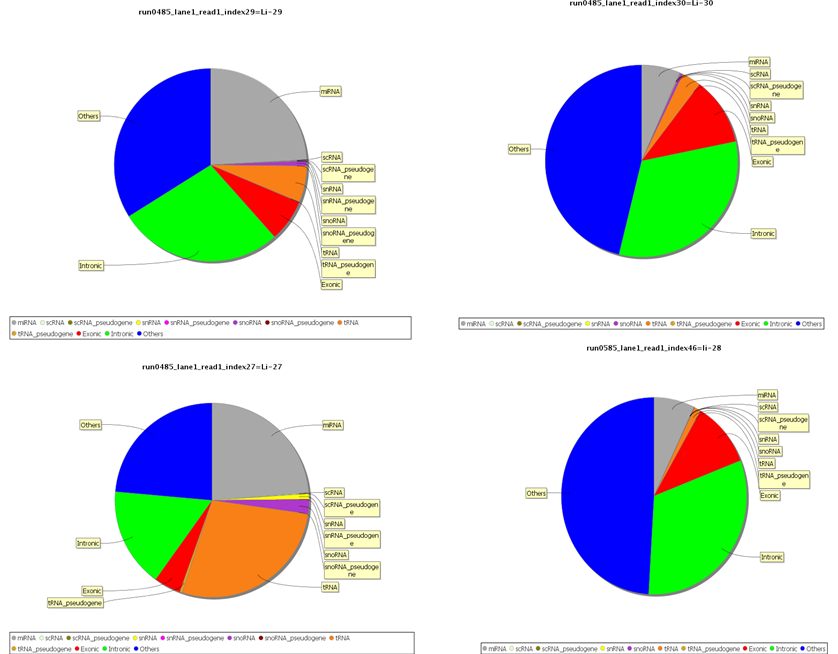


**S7.** Mitochondria-enriched miRNAs in 206 ρ^o^ cells and in 143B cells. Li-28=206 ρ^o^ mt-R RNA; Li-35=206 ρ^o^ mt RNA; Li-30=143B mt-R RNA; Li-29=143B C-p RNA.


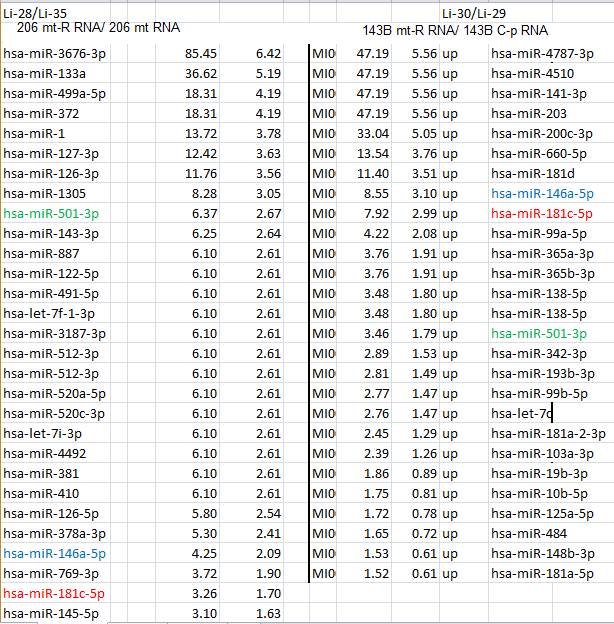


**S8.** Low-expressed miRNAs in 206 ρ^o^ mitochondria. The bottom was lowest expressed miRNA in 206 ρ^o^ mitochondria.


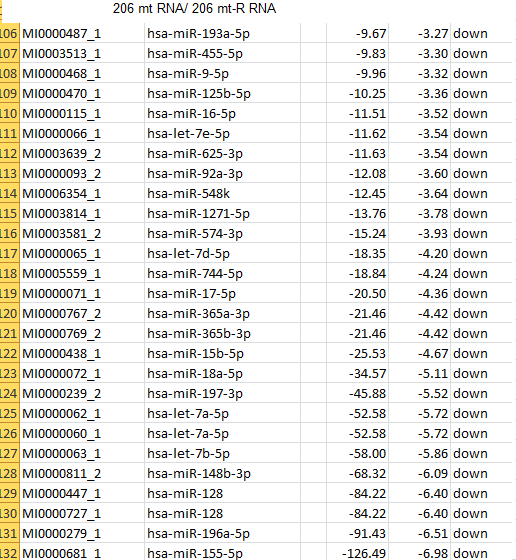


**S9.** PCR primers for checking mtDNA copy numbers.

HV1-f CAC CAT TAG CAC CCA AAG CT

HV1-r TGA TTT CAC GGA GGA TGG TG

H3-f GGAGAGGGCGTAAATTGTCT

H3-r GAGTGAACAGAGGTTCCAGTTT

**S10.** The RT-PCR and RT-qPCR primers for checking mitochondrial RNAs. 5S rRNA*,* encoded by nuclear gene and imported into mitochondria, was as control.


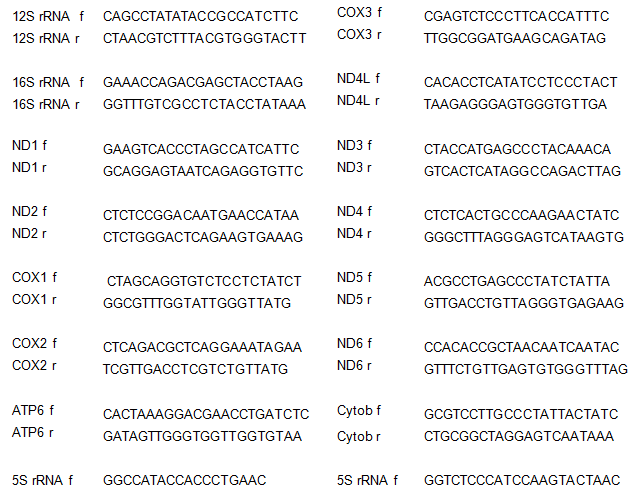

Supplement: Supplementary Information [file cddiscovery20154-s1.doc]
